# Correction: Chang et al. Decursinol Angelate Arrest Melanoma Cell Proliferation by Initiating Cell Death and Tumor Shrinkage via Induction of Apoptosis. *Int. J. Mol. Sci.* 2021, *22*, 4096

**DOI:** 10.3390/ijms23031629

**Published:** 2022-01-31

**Authors:** Sukkum Ngullie Chang, Imran Khan, Chang Geon Kim, Seon Min Park, Dong Kyu Choi, Heejin Lee, Buyng Su Hwang, Sun Chul Kang, Jae Gyu Park

**Affiliations:** 1 Advanced Bio Convergence Center (ABCC), Pohang Technopark Foundation, Pohang 37668, Korea; sukkumchang@gmail.com (S.N.C.); rjs6538@naver.com (C.G.K.); seonmin@ptp.or.kr (S.M.P.); 2 Department of Biotechnology, Daegu University, Gyeongsan 38453, Korea; imranakhan7@gmail.com; 3 The Hormel Institute, University of Minnesota, Austin, MN 55912, USA; 4 New Drug Development Center, DGMIF, 88 Dongnae-ro, Dong-gu, Daegu 41061, Korea; dongkyu@dgmif.re.kr (D.K.C.); jini150117@gmail.com (H.L.); 5 Nakdonggang National Institute of Biological Resources, Sangju 37242, Korea; hwang1531@nnibr.re.kr

The author wishes to make the following correction to this paper [1]:

In the original publication, there was a mistake in Figure 2. During manuscript preparation, the Figure 2B CDK4 blot was erroneously duplicated with CDK2. The authors did minor changes in statistical analysis in corresponding western blot quantification. These changes do not affect the conclusion and findings of the research article. The corrected Figure 2 and legend appears below.

The authors apologize for any inconvenience caused and state that the scientific conclusions are unaffected. The original publication has also been updated.

## Figures and Tables

**Figure 2:**
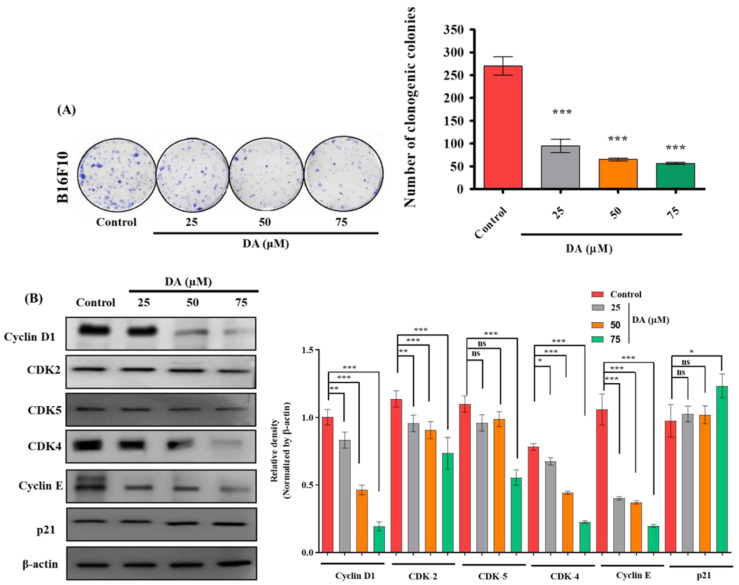
(**A**) Clonogenic assay and quantification of B16F10 cells cultured in the presence and absence of DA over 7 days, followed by crystal violet staining. (**B**) DA was treated to B16F10 cells for 24 h and cell cycle protein levels were detected, such as cyclin D1, CDK2, 5, 4, cyclin E, and p21. Densitometry analysis of the respective proteins was evaluated by Image J software, and results were normalized with β-actin. The data are represented as the means ± standard deviation (SD) of three independent experiments; ns—non-significant; * *p* < 0.05, ** *p* < 0.01, *** *p* < 0.001 vs. control, calculated through ANOVA prism.

## References

[1] Chang S.N., Khan I., Kim C.G., Park S.M., Choi D.K., Lee H., Hwang B.S., Kang S.C., Park J.G. (2021). Decursinol Angelate Arrest Melanoma Cell Proliferation by Initiating Cell Death and Tumor Shrinkage via Induction of Apoptosis. Int. J. Mol. Sci..

