# Supplementary material for: Decursinol Angelate Arrest Melanoma Cell Proliferation by Initiating Cell Death and Tumor Shrinkage via Induction of Apoptosis
Source: Int J Mol Sci. 2021 Apr 15;22(8):4096. doi: 10.3390/ijms22084096 (PMC8071397; doi:10.3390/ijms22084096)
Supplement: Supplementary file 1 [file ijms-22-04096-s001.zip › ijms-1164415-supplementary.pdf]

Supplementary Table 1. List of antibodies used in the study.

S.No	Protein name	Company name	Molecular weight	Dilution	Host	Secondary antibody
1.	p-JNK 1/2/3 (BS9939M)	Bioworld	46,54	1:1000	Rabbit	Goat anti rabbit-HRP
2.	Cyclin D1 (sc-753)	Santa Cruz	37	1:1000	Rabbit	Goat anti rabbit-HRP
3.	CDK2 (sc-163)	Santa Cruz	34	1:1000	Rabbit	Goat anti rabbit-HRP
4.	CDK4 (sc-260)	Santa Cruz	34	1:1000	Rabbit	Goat anti rabbit-HRP
5.	CDK5 (2506S)	Cell Signaling	30	1:1000	Rabbit	Goat anti rabbit-HRP
6.	Cyclin E (sc-481)	Santa Cruz	53	1:1000	Rabbit	Goat anti rabbit-HRP
7.	p21 (sc-397)	Santa Cruz	21	1:1000	Rabbit	Goat anti rabbit-HRP
8.	mTOR (sc-8319)	Santa Cruz	289,211-245	1:1000	Rabbit	Goat anti rabbit-HRP
9.	p-mTOR (sc-101738)	Santa Cruz	220	1:1000	Rabbit	Goat anti rabbit-HRP
10.	ATG-5 (12994S)	Cell Signaling	55	1:1000	Rabbit	Goat anti rabbit-HRP
11.	ATG-7 (8558S)	Cell Signaling	78	1:1000	Rabbit	Goat anti rabbit-HRP
12	Beclin-1 (3495S)	Cell Signaling	60	1:1000	Rabbit	Goat anti rabbit-HRP
13	LC-3A/B (L7543)	Santa Cruz	14-16	1:1000	Rabbit	Goat anti rabbit-HRP
14	JNK (BS1544)	Bioworld	46,54	1:1000	Rabbit	Goat anti rabbit-HRP
15	Bax (2772S)	Cell Signaling	20	1:1000	Rabbit	Goat anti rabbit-HRP
16	Bcl-2 (5114S)	Cell Signaling	28	1:1000	Rabbit	Goat anti rabbit-HRP
17	Cytochrome C (11940)	Cell Signaling	14	1:1000	Rabbit	Goat anti rabbit-HRP
18	CL Caspase 3 (BS4301)	Bioworld	22	1:1000	Rabbit	Goat anti rabbit-HRP
19	Caspase 9 (AB0816)	Bioworld	35,37,48	1:1000	Rabbit	Goat anti rabbit-HRP
20	PARP-1 (sc-7150)	Santa Cruz	90	1:1000	Mouse	Goat anti mouse-HRP
21	β-actin (#4967)	Cell signaling	45	1:1000	Rabbit	Goat anti rabbit-HRP
22	Secondary goat anti rabbit-HRP (NBP2-30348H) antibody	Novus Biologicals (Bethyl)	-	1:5000	Goat	-
23	Secondary donkey anti goat-HRP (NBP2-68552) antibody	Novus Biologicals (Bethyl)	-	1:5000	Donkey	-
24	Secondary goat anti mouse-HRP antibody	Santa Cruz	-	1:2000	Goat	-

25	Secondary sheep anti-rat-HRP (NBP2-22109) antibody	Novus Biologicals (Bethyl)	-	1:5000	Sheep	-

……

Supplementary Figures. Original uncropped images of western blots used for the experiment.

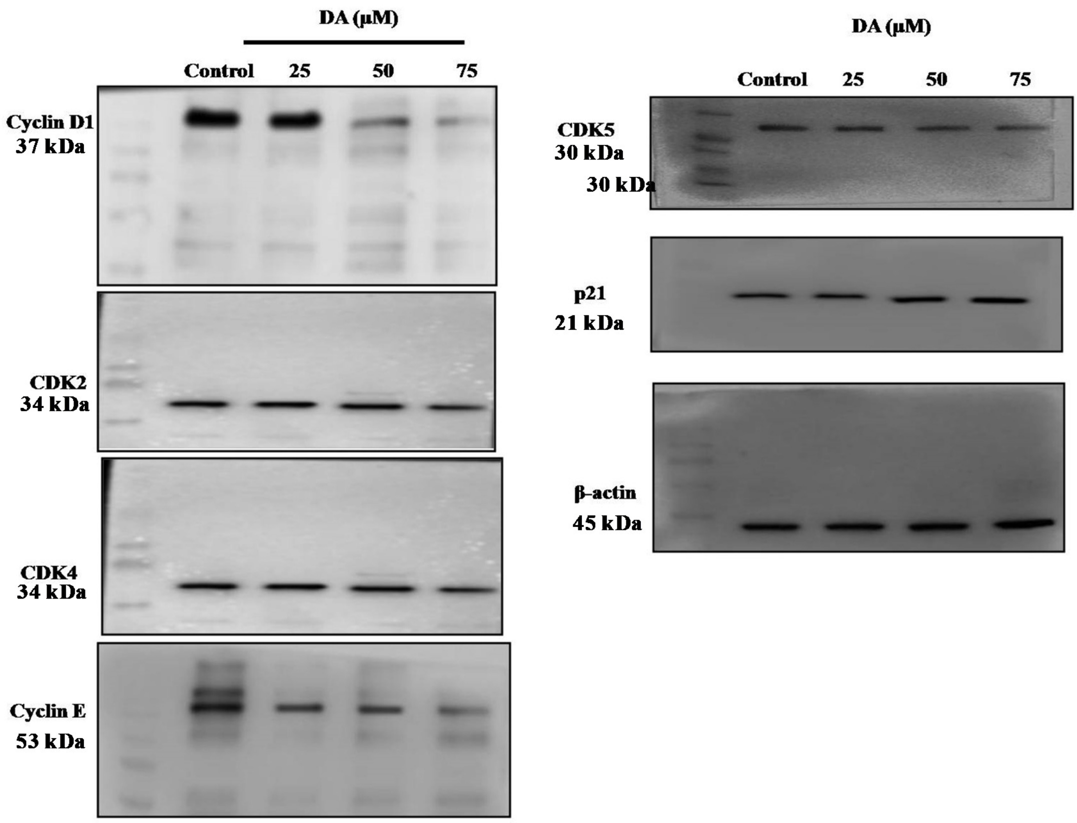

Fig 2.

…………………………………………………………………………………………………

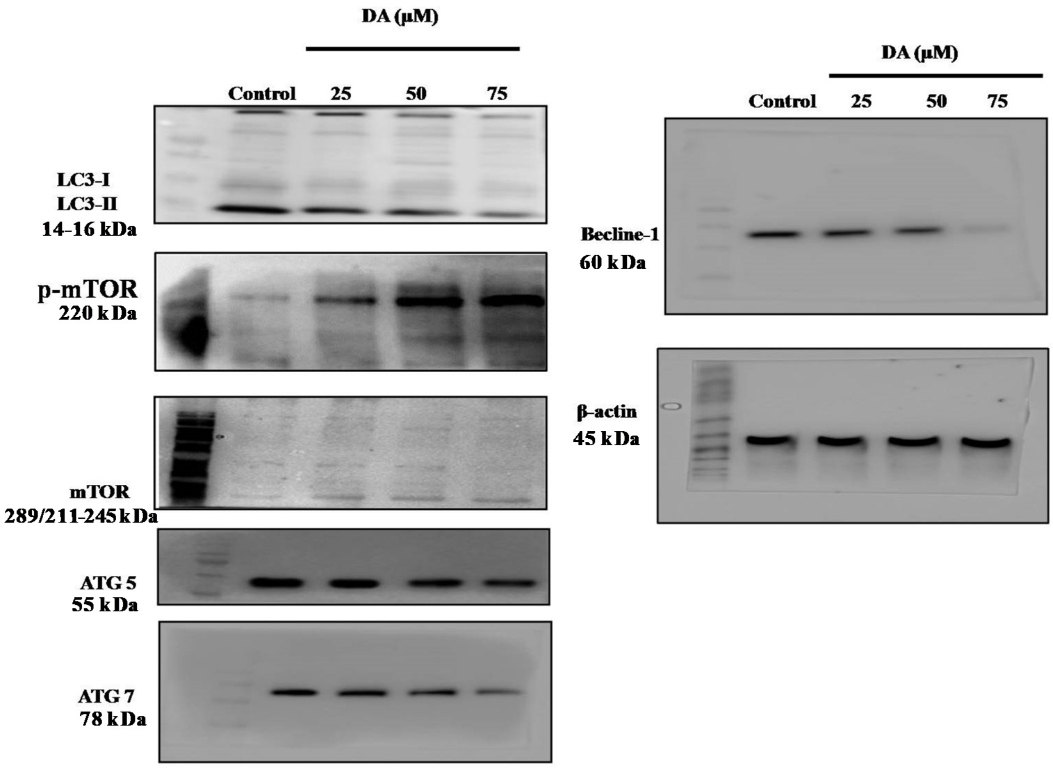
DA (µM)
Control
25
50
75
LC3-I
LC3-II
14-16 kDa
p-mTOR
220 kDa
mTOR
289/211-245 kDa
ATG 5
55 kDa
ATG 7
78 kDa
DA (µM)
Control
25
50
75
Becline-1
60 kDa
β-actin
45 kDa

Fig 3.

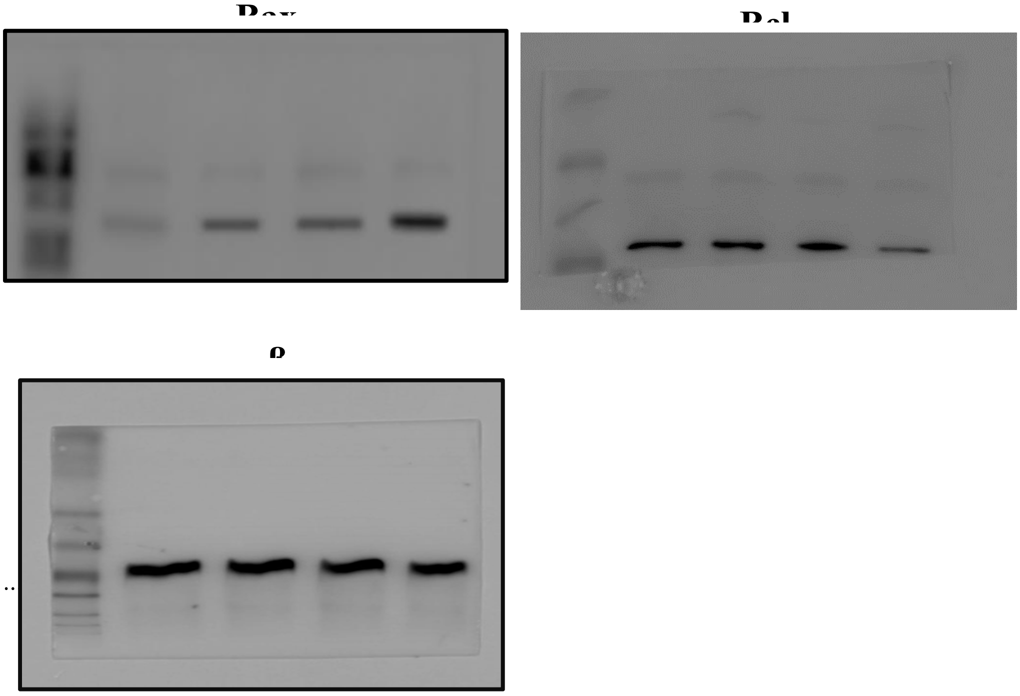

Fig. 4

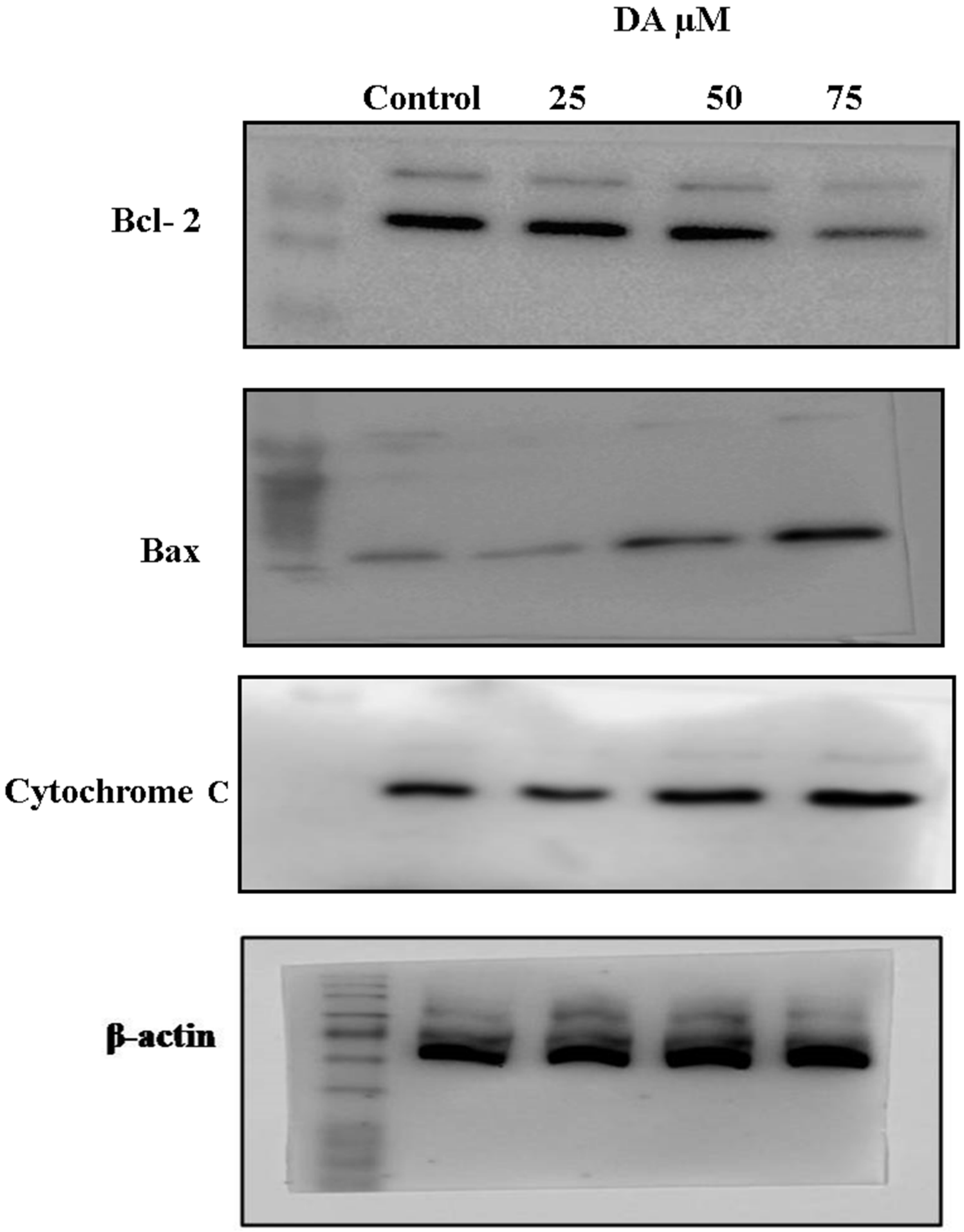

Fig. 6

……………………………………………………………………………………………

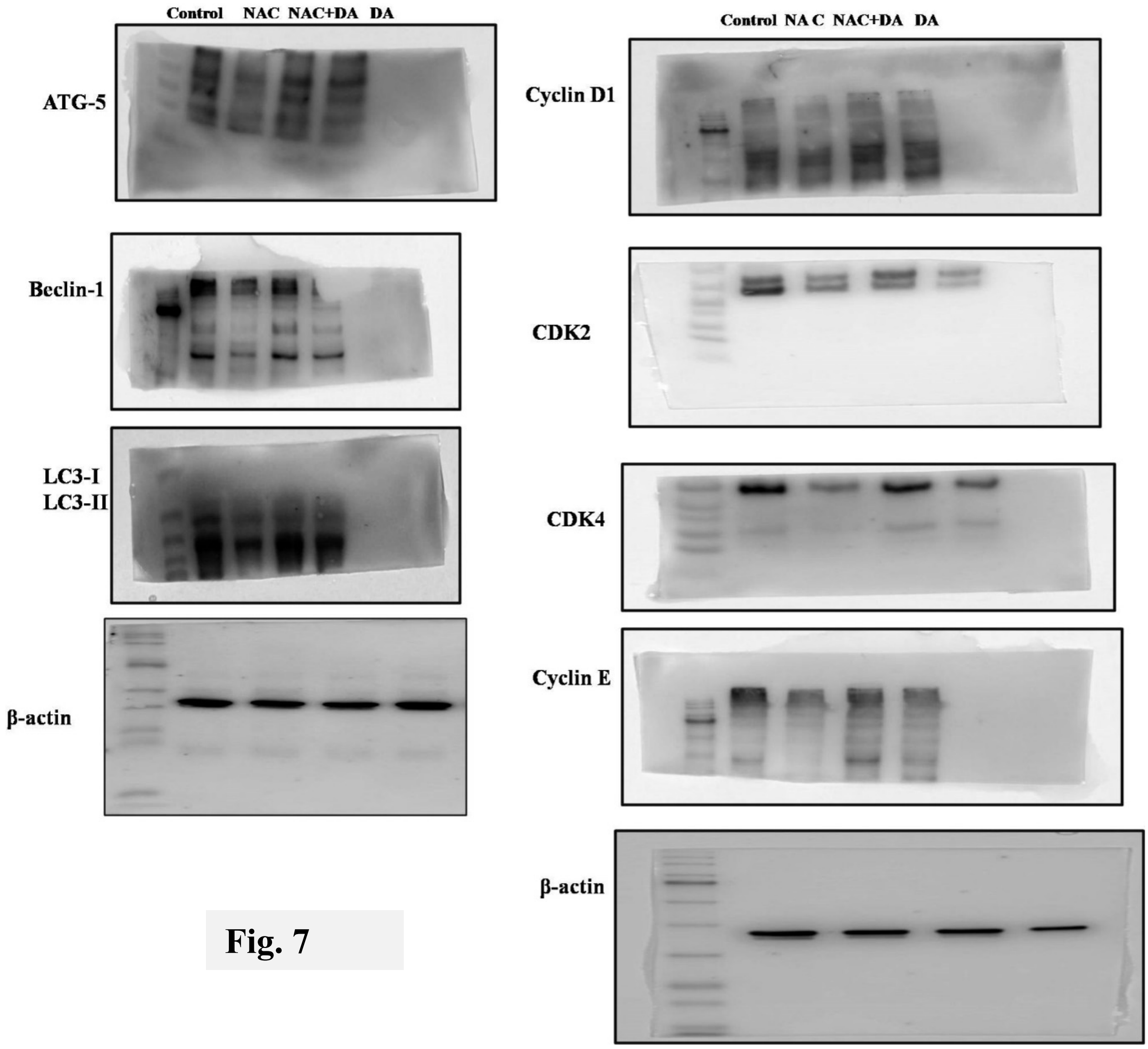
Control NAC NAC+DA DA
ATG-5
Beclin-1
LC3-I
LC3-II
β-actin
Control NA C NAC+DA DA
Cyclin D1
CDK2
CDK4
Cyclin E
β-actin

Fig. 7

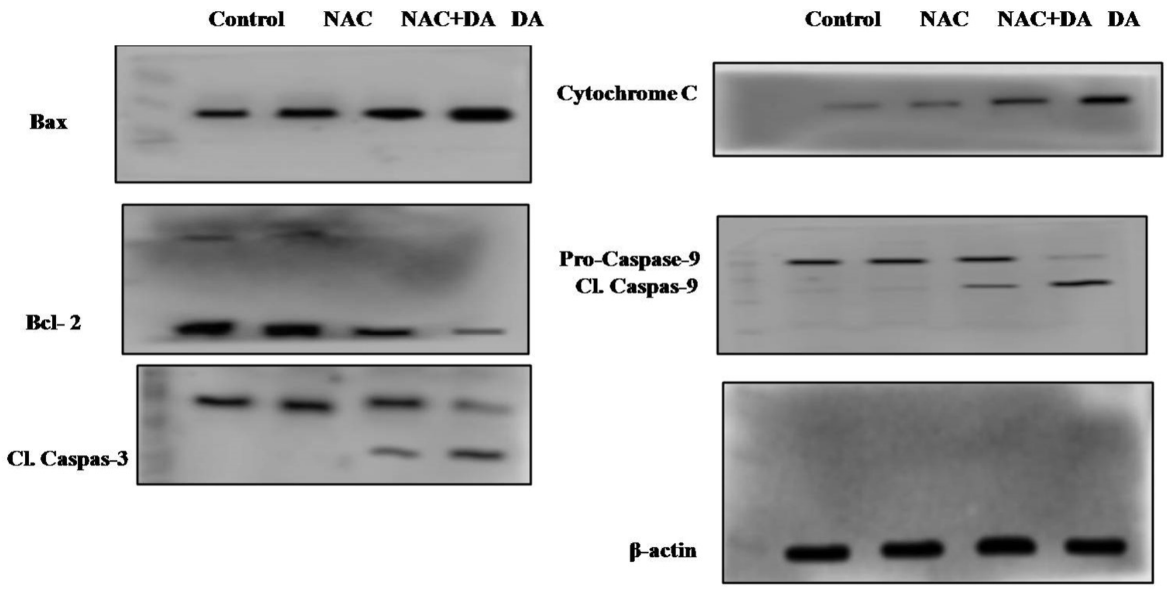
Control NAC NAC+DA DA
Bax
Bcl- 2
Cl. Caspas-3
Control NAC NAC+DA DA
Cytochrome C
Pro-Caspase-9
Cl. Caspas-9
β-actin

Fig. 8

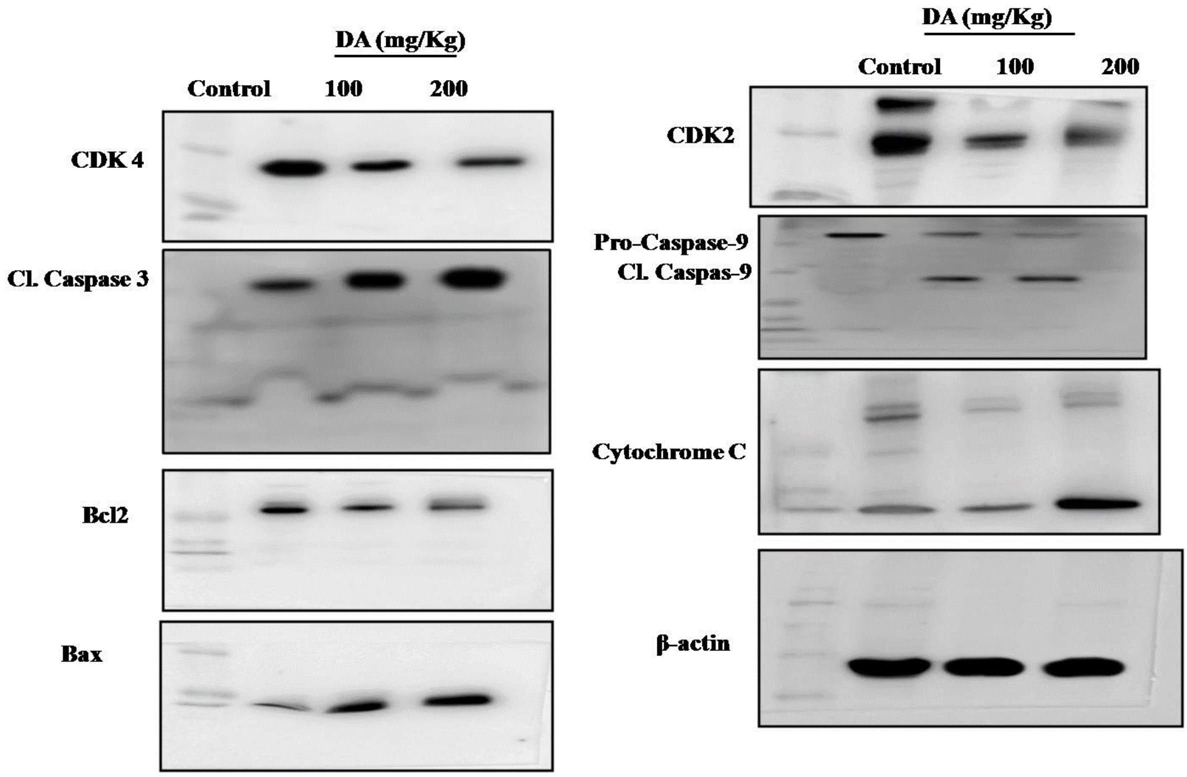
DA (mg/Kg)
Control 100 200
CDK 4
Cl. Caspase 3
Bcl2
Bax
DA (mg/Kg)
Control 100 200
CDK2
Pro-Caspase-9
Cl. Caspas-9
Cytochrome C
β-actin

Fig. 10
